# HP1a Recruitment to Promoters Is Independent of H3K9 Methylation in *Drosophila melanogaster*

**DOI:** 10.1371/journal.pgen.1003061

**Published:** 2012-11-15

**Authors:** Margarida L. A. Figueiredo, Philge Philip, Per Stenberg, Jan Larsson

**Affiliations:** 1 Department of Molecular Biology, Umeå University, Umeå, Sweden; 2 Computational Life Science Cluster (CLiC), Umeå University, Umeå, Sweden; University of Cambridge, United Kingdom

## Abstract

Heterochromatin protein 1 (HP1) proteins, recognized readers of the heterochromatin mark methylation of histone H3 lysine 9 (H3K9me), are important regulators of heterochromatin-mediated gene silencing and chromosome structure. In *Drosophila melanogaster* three histone lysine methyl transferases (HKMTs) are associated with the methylation of H3K9: Su(var)3-9, Setdb1, and G9a. To probe the dependence of HP1a binding on H3K9me, its dependence on these three HKMTs, and the division of labor between the HKMTs, we have examined correlations between HP1a binding and H3K9me patterns in wild type and null mutants of these HKMTs. We show here that Su(var)3-9 controls H3K9me-dependent binding of HP1a in pericentromeric regions, while Setdb1 controls it in cytological region 2L:31 and (together with POF) in chromosome 4. HP1a binds to the promoters and within bodies of active genes in these three regions. More importantly, however, HP1a binding at promoters of active genes is independent of H3K9me and POF. Rather, it is associated with heterochromatin protein 2 (HP2) and open chromatin. Our results support a hypothesis in which HP1a nucleates with high affinity independently of H3K9me in promoters of active genes and then spreads via H3K9 methylation and transient looping contacts with those H3K9me target sites.

## Author Summary

HP1 is a key protein in heterochromatin and epigenetic silencing, a phenomenon involving chromatin condensation. It is generally accepted that HP1 forms a dimer that links two adjacent nucleosomes through interactions with histone 3 methylated at lysine 9 (H3K9me). Since HP1 also interacts with the histone lysine methyltransferases (HKMTs) generating this modification, histone H3 becomes methylated and HP1 spreading is propagated. Here, we show that HP1a in *Drosophila* binds to promoters of active genes on chromosome 4 and pericentromeric regions. In contrast to current dogma, this binding is independent of H3K9me. In the presence of the HKMTs and H3K9me, HP1a is also enriched within the bodies of the bound genes. These findings shed new light on the role of HP1a and the epigenetic nature of this chromatin mark. We propose that HP1a interacts independently of H3K9me with the nucleosome with high affinity, probably via the H3 histone-fold. This interaction is followed by a more transient interaction between HP1a and H3K9me, which results in spreading of the HP1a enrichment into gene bodies. Overall, the presented results and hypothesized model provide an explanation for this epigenetic mark and possibly more general insights into the relationships between chromo-domain proteins and methylated histones.

## Introduction

Genomic DNA in eukaryotes is organized into chromatin, which has historically been divided into two distinct forms, euchromatin and heterochromatin, based on histological staining patterns. Euchromatin is defined as being decondensed during interphase while heterochromatin remains condensed throughout the cell-cycle, as reviewed in [1]. Euchromatin and heterochromatin can be distinguished by distinct histone modifications, one of which (H3 lysine 9 methylation, H3K9me) is generally associated with transcription repression and heterochromatin, or “green chromatin” according to recent, more elaborate chromatin structure-based definitions [2] used here. Three H3K9-specific methyl transferases (HKMTs) have been described in *Drosophila*: Su(var)3-9, Setdb1 and G9a. *Setdb1* (also known as *eggless*) plays an important role in oogenesis and its loss leads to female sterility [3]–[5]. *Su(var)3-9* and *G9a*, in contrast, are not essential for viability and their role during development appears to be less specific [6]–[9]. In addition to playing different roles during development the three HKMTs have been ascribed different regional properties. Chromosome staining experiments have shown that levels of pericentric H3K9me are reduced, but the enrichment of H3K9me on the 4^th^ chromosome remains unaltered in *Su(var)3-9* mutants [8], [10], [11]. Similar experiments on *Setdb1* mutants have shown that Setdb1 controls H3K9 methylation on the 4^th^ chromosome [12], [13]. However, the roles and specificity of G9a remains unclear, as does the redundancy of the three HKMTs [6], [7], [9].

The H3K9me-enriched heterochromatin, or more specifically the “green chromatin”, is also enriched in a number of specialized proteins [2], [14]. The pivotal protein for defining the “green chromatin” enriched in pericentromeric regions and on the 4^th^ chromosome is HP1a (heterochromatin protein 1, also known as Su(var)2-5). It is generally accepted that H3K9me stabilizes the binding of HP1a to chromatin [15]–[18]. The prevailing model postulates that HP1a forms a dimer through its C-terminal chromo-shadow domain and the two chromo-domains of the dimer link two adjacent nucleosomes through interactions with H3K9me. As HP1a interacts with the HKMTs (Su(var)3-9 or Setdb1), nearby H3K9 becomes methylated and HP1a spreading is propagated [19]. The link between H3K9me and HP1a is the most frequently described HP1a-chromatin interaction, but HP1 has also been shown to bind nucleosomes independent of the H3 tail [20] and with high affinity to the H3 histone-fold although the *in vivo* relevance of this binding is not known [21]–[24].

HP1a- and H3K9me-enriched chromatin is present in the pericentromeric regions as well as along the entire length of the small fourth chromosome in *Drosophila melanogaster*. Interestingly, despite the 4^th^ chromosome's repressive nature, with heterochromatic markers and large blocks of repeated sequences and transposed elements, genes on this chromosome are expressed with similar strength, or even more strongly, on average than genes on the other chromosomes [25], [26]. This can be partly explained by the fact that the 4^th^ chromosome has a unique chromosome-specific dosage compensating system mediated by the protein Painting of fourth (POF) [27]–[31]. POF is a protein that specifically targets active genes on the 4^th^ chromosome, binds their nascent RNA and mediates increases in their expression [26], [27], [32], [33]. Flies can survive without one copy of their 4^th^ chromosome or POF. However, haplo-fourth flies die if they lack POF [27]. POF has been shown to counteract the repressive influence on chromosome 4 genes caused by the heterochromatic nature of the chromosome [26], [27], [33]. The 4^th^ chromosome in *D. melanogaster* is therefore regarded as a good model for studying gene expression in heterochromatic environments [31], [34], [35]. Notably in this context, despite its name HP1a binds to active genes on the 4^th^ chromosome. On this chromosome an RNAi mediated knock-down of *HP1a* is associated with increased gene expression in adult flies [27], but there are also reported examples of *HP1a* loss or reduction being associated with decreased transcription [36]–[39].

High-resolution chromosomal distributions of HP1a [2], [14], [32], [35], [40]–[42] and POF [32] have been previously reported, but the dependence of HP1a binding on H3K9me2/3 and the different HKMTs at higher resolution has remained elusive. Therefore, to study the division of labor between the HKMTs and the dependence of HP1a binding on H3K9 methylation and POF *in vivo*, we have generated and analyzed high resolution H3K9me2, H3K9me3 and HP1a ChIP-chip profiles in wild type flies, null mutants of the three HKMTs and *Pof* mutants.

Our results show that Su(var)3-9 is mainly responsible for HP1a H3K9me-dependent binding to the pericentromeric regions while Setdb1 is responsible for HP1a H3K9me-dependent binding to cytological region 2L:31 and (together with POF) chromosome 4. HP1a binds to the promoters and within bodies of active genes in these three regions. More importantly, HP1a binding at promoters of active genes is independent of H3K9me and POF. Our results supports a model in which HP1a nucleates with high affinity independently of H3K9me and then spreads via transient looping contacts with H3K9me target sites.

## Results

### HP1a profiles in HKMT mutants

**Figure 1:**
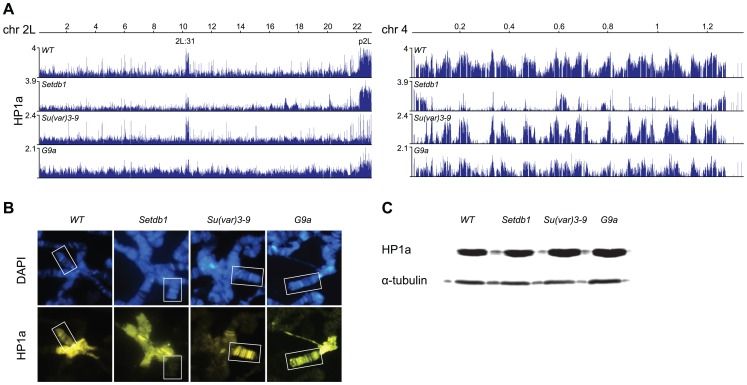
HP1a profiles in HKMT mutants. (A) HP1a binding profiles for the entire chromosome arm 2L and the 4^th^ chromosome in salivary gland tissue from wild type and null *Su(var)3-9*, *Setdb1* and *G9a* mutants. p2L and 2L:31 indicate the centromere proximal heterochromatin in chromosome 2L and the HP1a-enriched cytogenetic region in the middle of the chromosome arm, respectively. Numbers along the x-axis denote chromosomal positions along the chromosomes in Mb. The y-axis shows the ChIP enrichment in log_2_ ratios. (B) Immunostaining of polytene chromosomes from wild type and *Su(var)3-9*, *Setdb1* and *G9a* mutants with DAPI (blue) and HP1a (yellow). The fourth chromosomes are indicated by boxes. Note that HP1a is decreased on the 4^th^ chromosome in *Setdb1* mutants and in the pericentromeric heterochromatin in *Su(var)3-9* mutants. (C) Western blot showing total HP1a and tubulin from dissected salivary glands from wild type and *Su(var)3-9*, *Setdb1* and *G9a* mutants.

To study the dependence of HP1a binding on the three HKMTs in *Drosophila* we performed ChIP-chip analysis on chromatin extracts from salivary glands of 3^rd^ instar larvae, comparing wild type profiles to those of *Su(var)3-9*, *Setdb1* and *G9a* mutants (Figure 1A). It has previously been documented that HP1a binds to pericentromeric heterochromatin, chromosome 4 and part of cytological section 2L:31 [35], [36], [42]–[44]. Our generated ChIP-chip profiles confirm these observations: HP1a is enriched in region 2L:31, in the pericentromeric regions as exemplified by p2L (proximal 2L), and on chromosome 4 (Figure 1A, Figure S1). In *Setdb1* mutants we observed a strong reduction of HP1a on the 4^th^ chromosome and in region 2L:31, but region p2L appeared to be unaffected. In contrast, in *Su(var)3-9* mutants we observed a strong reduction of HP1a in p2L, but the 4^th^ chromosome and 2L:31 region were unaffected. In the *G9a* mutants we observed no regional or chromosome-specific reduction. Note that any potential global increase or decrease in enrichment is difficult to measure in ChIP-chip experiments since the dynamic range of ChIPs typically varies substantially between experiments.

Results from polytene chromosome stainings of HP1a in wild type and the three HKMT mutants (performed to confirm the results and look for global changes) were in good agreement with the ChIP-chip profiles. As can be seen in Figure 1B, HP1a is reduced on the 4^th^ chromosome in *Setdb1* mutants compared to wild type and in the pericentromeric regions in *Su(var)3-9* mutants. No obvious difference in HP1a binding was observed between wild type and *G9a* mutants (Figure 1B). Using Western blot analysis we observed no clear differences in total amounts of HP1a protein between wild type and the three HKMT mutants (Figure 1C). We conclude that HP1a binding on the 4^th^ chromosome and region 2L:31 depends on Setdb1, HP1a binding in pericentromeric regions depends on Su(var)3-9 while we found no clear dependence of its binding on G9a in our experiments.

### *Drosophila* HKMTs have region-specific functions in generating H3K9me

**Figure 2:**
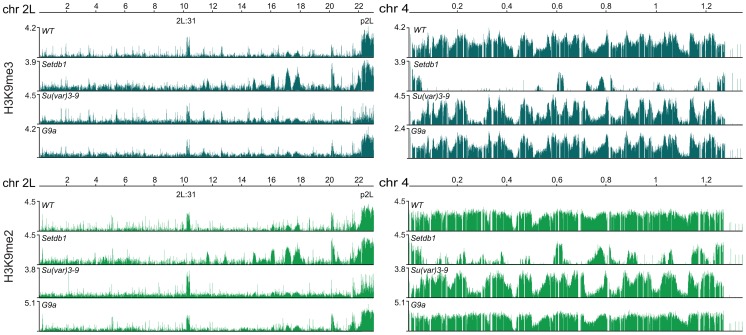
H3K9 methylation profiles in HKMT mutants. H3K9me3 (gray) and H3K9me2 (green) profiles for the entire chromosome arm 2L and the 4^th^ chromosome in salivary gland tissue from wild type and null *Su(var)3-9*, *Setdb1* and *G9a* mutants. p2L and 2L:31 indicate the centromere proximal heterochromatin in chromosome 2L and the HP1a-enriched cytogenetic region in the middle of the chromosome arm, respectively. Numbers along the x-axis denote chromosomal positions along the chromosomes in Mb. The y-axis shows the ChIP enrichment in log_2_ ratios. Note that both H3K9me2 and H3K9me3 are strongly reduced in region 2L:31 and chromosome 4 in *Setdb1* mutants, and strongly reduced in p2L in *Su(var)3-9* mutants.

H3K9me2 and H3K9me3 are documented targets for HP1a binding [15]–[18]. We therefore tested the dependence of these two modifications on each of the three HKMTs. The results show that H3K9me2 was strongly reduced in region 2L:31 and the 4^th^ chromosome, while virtually all H3K9me3 was lost in region 2L:31 and strongly reduced on chromosome 4 in *Setdb1* mutants (Figure 2). In *Su(var)3-9* mutants both H3K9me2 and H3K9me3 were strongly reduced in the pericentromeric regions. Importantly, in both *Su(var)3-9* and *Setdb1* mutant backgrounds, the H3K9me3 reductions were more dramatic than the corresponding H3K9me2 reductions, although the affected regions differed between the mutants. In the *G9a* mutants we detected no specific effects on either H3K9me2 or H3K9me3 (Figure 2). Comparing the HP1a binding and H3K9me profiles on the 4^th^ chromosome we observed regions that were not dependent on Setdb1. These regions will be described in more detail later. Since the overall HP1a, H3K9me2 and H3K9me3 profiles in *G9a* mutants were not altered compared to wild type we concentrated our analysis on *Setdb1* and *Su(var)3-9*. We conclude that Setdb1 and Su(var)3-9 methylates H3K9 in different regions and loss of Su(var)3-9 or Setdb1 affects both H3K9me2 and H3K9me3, but in different regions of the genome.

### HP1a binds promoters independently of methylation

**Figure 3:**
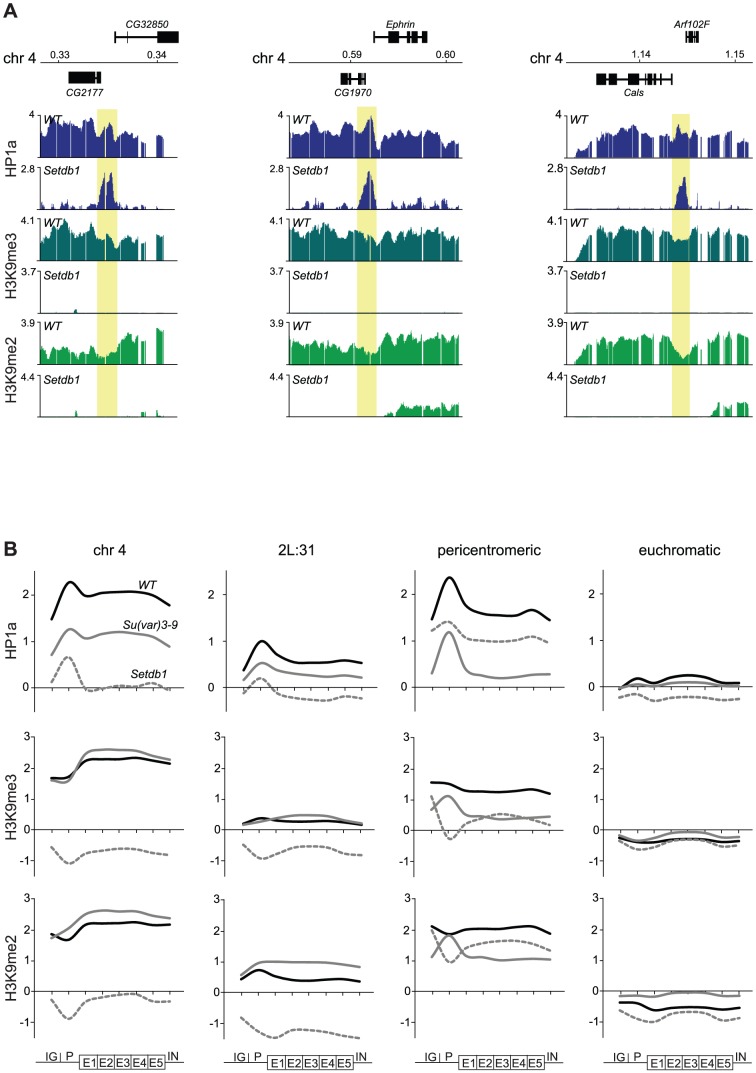
HP1a binds independently of H3K9me to promoters and spreads H3K9me-dependently into gene bodies. (A) HP1a and H3K9me profiles in three illustrative regions of the 4^th^ chromosome in wild type and *Setdb1* mutant backgrounds. Numbers along the x-axis denote chromosomal positions along the chromosomes in Mb. The y-axis shows the ChIP enrichment in log_2_ ratios. Genes expressed from left to right and vice versa are shown above and below the horizontal lines, respectively. The HP1a methylation-independent promoter peaks are indicated by yellow boxes (B) Average metagene profiles of HP1a, H3K9me2 and H3K9me3 on the 4^th^ chromosome, region 2L:31,the pericentromeric regions and the euchromatic regions, based on eight enrichment values for each active gene in the respective regions (x-axis). The y-axis shows the ChIP enrichment in log_2_ ratios. The first points (IG) of the curves show average values for the intergenic regions upstream of the designated promoters of the genes. The promoter (P) is defined as the 500 bp region upstream of the TSS. The gene bodies are divided into five bins (E1–E5) and the average enrichment in introns (IN) is indicated by the last point of each curve. The average profiles for wild type (solid black), *Su(var)3-9* mutants (solid gray) and *Setdb1* mutants (dashed gray) are shown.

We have previously shown that HP1a binds not only within bodies of expressed genes on the 4^th^ chromosome but also in a distinct peak in the promoter of most targeted genes [32]. Interestingly, in the present study we observed several HP1a peaks on the 4^th^ chromosome and region 2L:31 of *Setdb1* mutants that did not correspond to H3K9me. This can be readily seen by comparing the HP1a and H3K9me profiles for *Setdb1* (Figure 1A and Figure 2, respectively, i.e., regions that lack H3K9me and retain HP1a in the *Setdb1* mutant background). We therefore explored the locations of these “methylation-independent” HP1a peaks, and found them to be concentrated in the promoters of genes, which importantly contain no detectable H3K9me (Figure 3A and Figure S2). To analyze the binding further at a gene feature level we generated average gene profiles of all active genes in the four defined regions (chromosome 4, pericentromeric regions, euchromatic regions and 2L:31) as described in Materials and Methods. The average HP1a binding, H3K9me2 and H3K9me3 profiles (Figure 3B) show that HP1a within the gene bodies in specific regions is lost in mutants of the corresponding region-specific HKMTs. However, more importantly, the HP1a peaks in the promoters are resistant to the loss of H3K9me. Interestingly, H3K9me in pericentromeric promoters depends on Setdb1 and not on Su(var)3-9 (Figure 3B and Figure S2). Note that the enrichment of HP1a in the promoter peaks is reduced upon loss of methylation, but can still be readily seen (Figure 3A and 3B). These findings indicate that HP1a may bind to promoters independently of methylation, then further H3K9me-dependent binding occurs through interaction with the corresponding HKMT (Setdb1 for the 4^th^ chromosome and 2L:31 and Su(var)3-9 for the pericentromeric region). This model explains not only the observed promoter peaks but also spreading of the HP1a binding to the gene bodies. The HP1a enrichment within the gene bodies is most pronounced on the 4^th^ chromosome.

### HP1a-enriched promoters are A/T rich and enriched in HP2

**Figure 4:**
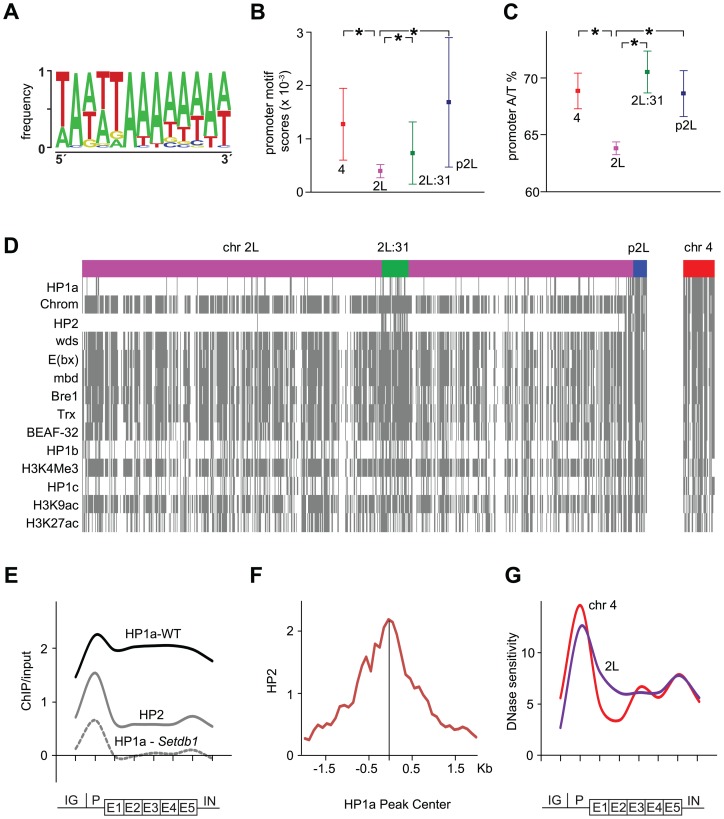
HP1a-enriched promoters are A/T rich, bound by HP2 and DNase sensitive. (A) Sequence motif over-represented in HP1a-enriched promoters. (B) Motif scores in promoters from chromosome 4 (red), 2L:31 (green), pericentromeric region (blue) and, as a control, chromosome 2L (pink). (C) A/T content in promoters. In (B) and (C) significant differences are indicated by * (Mann-Whitney U test, p<0.01). (D) Binary heat-map showing significant enrichment of indicated chromatin factors at promoters on chromosomes 2L and 4. Presence of a protein at a promoter is indicated by a gray line. Note the almost perfect correlation between HP1a and HP2. (E) Average metagene profiles showing the ChIP enrichments of HP1a in wild type (black), HP1a in *Setdb1* mutants (dashed gray) and HP2 in wild type (gray) on the 4^th^ chromosome (log_2_ ratios). (F) HP2 enrichment (log_2_ ratios) plotted around HP1a peak centers of 34 methylation-independent HP1a peaks. (G) Average metagene profiles of DNase sensitivity for the 4^th^ chromosome (red) and chromosome 2L (blue). The y-axis shows the density of mapped DNaseI cleavages in a 150 bp sliding window [82].

Intrigued by the methylation independence of HP1a in the promoters we further characterized HP1-enriched promoters by exploring possible correlations between them and both sequence composition and protein factors. To test for specific sequence motifs we defined HP1a promoter peaks for genes on the 4^th^ chromosome, region 2L:31 and p2L as 50 bp +/− each HP1a peak center and used these sequences as input in the CompleteMOTIFS platform. An A/T rich motif was found that was significantly enriched in promoters in chromosome 4, 2L:31 and pericentromeric regions compared to the rest of chromosome 2L (Figure 4A–4B). Motif scores were calculated by scanning the identified motif in promoters (500 bp upstream of TSS) in the 4^th^ chromosome, region 2L:31, p2L, and the remaining part of chromosome 2L as a control. A/T rich motifs have previously been shown to be enriched in HP1a binding sites [45]. Since the HP1a bound promoters were also found to be enriched with an A/T rich motif, we assessed the A/T content in these promoters, and found it to be significantly higher in the three HP1a-enriched regions than in promoters in the control chromosome region (Figure 4C).

Next, to seek protein factors and chromatin modifications that correlate with enriched HP1a levels in promoters we identified chromatin factors that were classified as bound (according to modENCODE classification) to more than 50% of the promoters of active genes on the 4^th^ chromosome. Out of 51 tested chromatin factors 14 were classified as bound to >50% of chromosome 4 promoters including, as expected, HP1a as the protein with highest overlap. A binary heat-map of these factors is shown in Figure 4D. As shown, HP1a is classified as bound to a number of promoters along chromosome 2L and concentrated in region 2L:31 and p2L. The promoters bound by HP1a are targeted by a number of other factors, e.g., Chromator, E(bx), Trx and BEAF-32. Most of these factors are not specific for the promoters bound by HP1a but bind to a large number of promoters (Figure 4D). However, heterochromatin protein 2 (HP2) binding shows a close to perfect correlation with HP1a bound promoters and is almost completely absent outside 2L:31 and p2L on chromosome 2L (Figure 4D and Figure S3). Furthermore, the gene average and enrichment profiles of HP2 show that the correlation between HP1a and HP2 is indeed concentrated in the promoters (Figure 4E and Figure S3). To further test the link between HP2 and the methylation-independent HP1a promoter peaks we manually annotated 34 confidently identified HP1a promoter peaks on the 4^th^ chromosome and plotted the HP2 enrichment in relation to their centers. The results show that the HP2 enrichment is significantly higher in these peaks than in other promoters (Figure 4E and 4F, Table S1) and that HP2 peaks in the annotated center of the HP1a peaks. We conclude that HP2 is strongly associated with the HP1a methylation-independent promoter peaks. Again, this is suggestive of a fundamental difference between HP1a binding to promoters and in gene bodies.

As its name implies, HP1a is thought to cause the formation of inaccessible, condensed chromatin. We therefore plotted average DNase sensitivity profiles, as a marker for accessible chromatin. This revealed that chromosome 4 promoters are more sensitive to DNase than chromosome 2L, despite the enriched HP1a levels in chromosome 4 promoters (Figure 4G). In contrast, within the gene bodies, chromosome 4 genes are slightly less sensitive. Interestingly, on the 4^th^ chromosome HP1a binds both to promoters and gene bodies, although the DNase sensitivity is still slightly lowered in gene bodies but increased in promoters compared to other genomic regions. These correlation-based findings support the hypothesis that HP1a binding to promoters and gene bodies differs in both mechanism and outcome.

### The gene body targeting but not the promoter targeting of HP1a depends on POF

**Figure 5:**
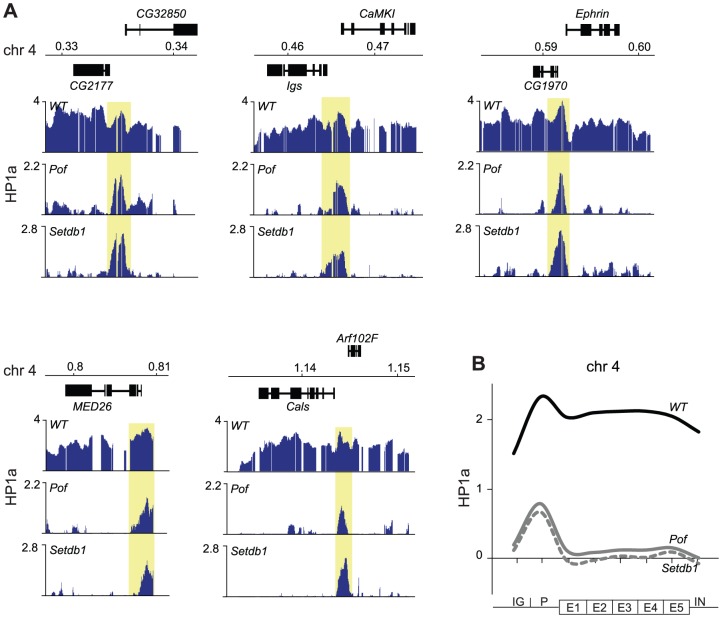
HP1a binds independently of POF and Setdb1 to promoters. (A) HP1a profiles in five illustrative regions of the 4^th^ chromosome in wild type, *Pof* and *Setdb1* backgrounds. Numbers along the x-axis denote chromosomal positions along the chromosomes in Mb. The y-axis shows the ChIP enrichment in log_2_ ratios. Genes expressed from left to right and vice versa are shown above and below the horizontal lines, respectively. The HP1a methylation-independent promoter peaks are indicated by yellow boxes. (B) Average metagene profiles of HP1a enrichments on the 4^th^ chromosome in wild type (solid black), *Pof* (solid gray) and *Setdb1* (dashed gray) backgrounds.

Considering the hypotheses that HP1a binds independently of methylation to promoters and this is followed by H3K9me-dependent spreading we were interested to identify factors that may be linked to the spreading. It has been previously shown that binding of HP1a to gene bodies is linked to active genes [32], [35], and we have shown by polytene chromosome stainings that binding of HP1a to the 4^th^ chromosome depends on POF [27]. To test the dependence of HP1a binding to this chromosome on POF at a gene level we performed ChIP-chip analyses of HP1a-DNA interactions in *Pof* mutants (Figure 5). Strikingly, the HP1a profiles of *Pof* and *Setdb1* mutants were very similar, i.e., the gene binding of HP1a was lost but the prominent HP1a promoter peaks were retained. We conclude that spreading of HP1a to the gene bodies depends on H3K9me generated by Setdb1 and the presence of POF protein. Furthermore, HP1a promoter peaks on the 4^th^ chromosome are independent of H3K9me and are resistant to losses of both Setdb1 and POF.

### The 4^th^ chromosome contains domains targeted by HP1a independently of *Pof* and *Setdb1*

**Figure 6:**
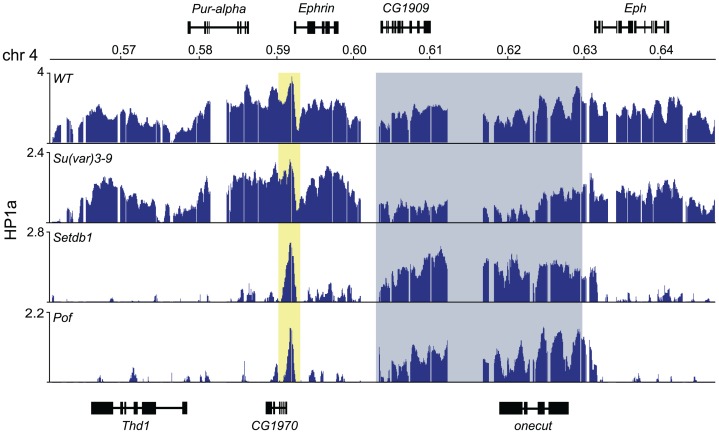
HP1a binds two unexpressed genes in the middle of the 4^th^ chromosome independently of Setdb1 and POF. HP1a enrichment profiles at a 90 kb region in the middle of the 4^th^ chromosome in wild type, *Pof* and *Setdb1* backgrounds. Numbers along the x-axis denote chromosomal positions along the chromosomes in Mb. The y-axis shows the ChIP enrichment in log_2_ ratios. Genes expressed from left to right and vice versa are shown above and below the horizontal lines, respectively. The peak within the yellow box corresponds to the *CG1970 Ephrin* promoter peak. The two genes *CG1909* and *onecut* within the gray box are unexpressed genes that bind HP1a independently of POF and Setdb1.

Analyzing the HP1a profile on the 4^th^ chromosome in wild type and HKMTs mutants we observed three regions that were resistant to the loss of Setdb1 (see Figure 1A). First, there is a region in the proximal part of the 4^th^ chromosome that retains HP1a in *Setdb1* mutants, covering the three pseudogenes *CR32009*, *CR320010* and *CR320011*. This region does not respond to *Setdb1* but responds to *Su(var)3-9*. Thus, the proximal region of the 4^th^ chromosome behaves similarly in this respect to the proximal region of the other chromosome arms, e.g., p2L. Secondly, in the most distal gene on the 4^th^ chromosome, *Caps*, HP1a binding is independent of Setdb1. Finally, there is a region in the middle of the chromosome (region 600–630 kb with two genes, *onecut* and *CG1909*) in which the HP1a binding is independent of Setdb1 and POF (Figure 6). The HP1a enrichment in these two genes is reduced in *Su(var)3-9* mutants but not lost. Interestingly, these two genes are not expressed in salivary glands, thus they represent two HP1a targeted, but unexpressed genes.

## Discussion

Chromosome 4 is considered to be a repressive environment that is enriched in heterochromatin markers such as HP1a and methylated H3K9 [8], [11], [35], [46]. It contains large blocks of repeated sequences and transposable elements interspersed with the genes [47]–[52], and transgenes inserted on the 4^th^ chromosome often show variegated expression because of partial silencing [53]–[56]. Despite its heterochromatic nature genes located on the 4^th^ chromosome are expressed as strongly on average, or even more strongly, than genes on other chromosomes [25], [26]. Traditionally, the division of genomes into heterochromatic and euchromatic regions was based on cytological characteristics of chromatin in interphase. Today more elaborate definitions of chromatin states are available based on chromatin components, such as the five principal chromatin types defined in [2]. According to these definitions, pericentromeric heterochromatin and the 4^th^ chromosome is highly enriched in “green-chromatin”. Similar definitions have been constructed by the modENCODE project, distinguishing nine chromatin states [14], one of which (chromatin state 7) corresponds to “green-chromatin”. HP1a and H3K9me2/me3 are the key components distinguishing green-chromatin and chromatin state 7. Our maps of HP1a and H3K9me2/me3 in chromatin from dissected salivary gland tissue correlate with previously reported high-resolution ChIP-chip and DamID maps of chromatin from various cell lines, embryos and fly heads [2], [14], [35], [40], [41]. Thus, the main regional chromosome organization into this chromatin type appears to be stable during development.

Our results show that the regional enrichment of HP1a depends on region-specificity of the HKMTs. The regional differences we observed in whole chromosomes confirm previous results based on chromosome stainings, i.e., loss of Su(var)3-9 causes a reduction of HP1a and H3K9me in pericentromeric regions but not the 4^th^ chromosome [8], [10], [11] while loss of Setdb1 causes reductions of HP1a and H3K9me on the 4^th^ chromosome and in region 2L:31 [12], [13]. Loss of G9a results in no difference in H3K9me, in accordance with previous reports [6]. We see no clear indications of redundancy between the different HKMTs.

The most important observations in our study are the fundamental differences between HP1a enrichment responses in gene bodies and promoters to losses of HKMT and H3K9 methylation. Upon loss of the region-specific HKMT, HP1a is strongly reduced or lost in gene bodies but the HP1a promoter peak is retained. These effects were observed in the pericentromeric region in *Su(var)3-9* mutants and both the 4^th^ chromosome and region 2L:31 in *Setdb1* mutants. The observed HP1a binding in promoters is strongly indicative of H3K9me-independent nucleation sites. Interestingly, although the interaction between HP1a and methylated H3K9 is well documented, and was confirmed in our experiments, HP1 proteins have been shown to bind only weakly to reconstituted methylated nucleosomal arrays and purified native chromatin [22], [57], [58]. For example, H3 peptides containing H3K9me3 bind HP1 with relatively weak (µM) affinity [18], [59]. This is in stark contrast to their nM affinity for unmodified histones [60] and the stable interaction of HP1, probably to the histone fold region of H3, that occurs in S phase when DNA replication disrupts the histone octamers [21], [22]. We conclude that HP1a binds to two distinct targets in chromatin: very stably and methylation-independently to promoters of active genes (probably via interactions within the nucleosomes) and less stably (but with perfect correlation) to methylated H3K9 sites.

Considering the methylation-dependent and -independent binding of HP1a it is interesting to note that HP1a is essential for viability [46], in contrast to the three studied HKMTs. *Su(var)3-9* is not required for viability and homozygous null mutant stocks can be kept. The same is true for *G9a* [6]. *Setdb1* is claimed to be essential in *Drosophila* [12] and is certainly required for female fertility [4]. Nevertheless, in uncrowded conditions *Setdb1^10.1a^* homozygous flies hatch although they have decreased viability (J. Larsson unpublished results), and pairwise crossings of the HKMT mutants have showed no clear effects in terms of reduced viability [6], [7]. Our findings of H3K9me-independent HP1a binding to promoters tempt speculation that HP1a may be essential for survival due to the methylation-independent promoter binding of HP1a. However, HP1a has also been associated with non-chromatin based functions such as linkage to hnRNP particles, suggesting it may also be involved in RNA compaction [38], although the importance of this function remains elusive.

The characterization of the HP1a bound promoter peaks led to two important findings. Firstly, promoters in the 4^th^ chromosome and region 2L:31 have a significantly higher A/T content compared to promoters at other chromosomal locations. The presence of A/T rich motifs in general HP1a target sites have previously been reported [45] and our results confirm that this is also true for promoter-specific HP1a targets. It has been shown that poly(dA:dT) tracts in promoters disfavours nucleosomes and modulate gene expression levels [61], [62]. In addition, promoters in the 4^th^ chromosome are more DNase sensitive than promoters at other genomic locations, suggesting that the chromatin structure is more open within these promoters. In fact, it has been proposed that HP1a promotes an open chromatin structure at bound promoters [63]. In contrast, gene bodies in the 4^th^ chromosome are slightly less accessible to DNase, which again indicates that the HP1a binding to promoters is fundamentally different to the HP1a targeting in gene bodies. The slightly reduced DNase sensitivity in gene bodies is also consistent with the previously observed reduction of transcription elongation efficiency of genes on the 4^th^ chromosome [26]. Secondly, in our search for chromatin-associated factors that correlate with the HP1a binding promoter peaks we found that HP2 shows a close to perfect correlation. HP2 has previously been shown to interact with HP1a [64]–[66], and it has been suggested that HP2 interaction with the HP1a chromo-shadow domain drives HP1a dimerization [64]. Our results show that the HP1a-HP2 interactions mainly occur at the HP1a bound promoters. This is consistent with previous observations that all mutations affecting HP1 dimerization abolish the interactions between HP1 and non-modified H3 [24], since these interactions should mainly occur at promoters according to our data.

Our results provide strong support for the model proposed by [22] that high affinity HP1a binding to the histone fold provides a nucleation site for HP1a targeting to chromatin. It is interesting to note that this incorporation is suggested to occur when the histone fold region of H3 becomes exposed because of active transcription, histone variant exchange or replication. A link between HP1 and replication has also been demonstrated by observed interactions between HP1 and the origin recognition complex (ORC) [67], [68], and the requirement of human ORC association with HP1 for correct targeting to heterochromatin [69]. In addition, HP1a modulates replication timing in *Drosophila* and reduced levels of HP1a result in delayed replication of chromosome 4 [39]. We speculate that HP1a binding to promoters avoids delay of this heterochromatic region's replication, that it provides an epigenetic nucleation mark for HP1a, and that the resulting nucleation is followed by a low affinity spreading to gene bodies. We envision a transient looping contact model in which the low affinity between HP1a and H3K9me provides the means for spreading of HP1a, analogous to the proposed model for the interactions of another *Drosophila* chromodomain protein, Polycomb (Pc). The chromo-domain of Pc interacts with H3K27me, but the nucleation sites for Pc are the Polycomb Response Elements, which have lower levels of H3K27me [70]. Thus, the nucleation appears to be independent of H3K27me and is followed by spreading caused by transient contacts between Pc and H3K27me [71], [72]. Similarly to HP1a and H3K9me, the affinity of the Pc chromo-domain to H3K27me is relatively weak, with a dissociation constant in the µM range [73]. In the case of HP1a the proposed spreading correlates with (and thus presumably depends on) at least three factors: H3K9me, active transcription and POF. The spreading appears to be generally restricted to transcribed genes, although there are two exceptions (*onecut* and *CG1909*) on the 4^th^ chromosome. On the 4^th^ chromosome, where POF binds to gene bodies, the HP1a enrichment is much higher than in region 2L:31. It should be stressed that HP1a and POF bindings are interdependent and POF also requires Setdb1 to target the 4^th^ chromosome [13], [27]. Thus, the relationships between these factors remain elusive. Why is the gene body targeting of HP1a on the 4^th^ chromosome *Pof*-dependent? This cannot be explained by expression differences, because although expression levels drop in *Pof* mutants the reductions are minor [26], [27], [33]. Our hypothesis is that POF binding to nascent RNA on active chromosome 4 genes may stabilize the interaction between HP1a and H3K9me as an adaptor system linking histone marks to nascent RNA, similar to the chromatin adaptor model for alternative splicing [74], [75].

The enrichment of H3K9me on the 4^th^ chromosome mainly depends on Setdb1, but in the most proximal region of the 4^th^ chromosome the H3K9me is Su(var)3-9 dependent. Thus, the proximal region of chromosome 4 is similar to the proximal region of other chromosome arms in this respect. Position-effect variegation studies have shown that although most variegated (partially silenced) transgenic inserts on the 4^th^ chromosome are suppressed in *Setdb1*, but not *Su(var)3-9* mutants, the reporter insertion 118E-10 is suppressed in *Su(var)3-9* mutants [7], [76]. Interestingly, this transgene is inserted in the pericentric region on the 4^th^ chromosome, i.e., the region that according to our study is dependent on Su(var)3-9.

In summary, we report dual binding properties of the HP1a protein: an H3K9me methylation-independent binding at promoters and a methylation-dependent binding within gene bodies suggested to occur by spreading. Like arms of other chromosomes, the proximal region of the 4^th^ chromosome is enriched in HP1a and Su(var)3-9-dependent H3K9me. However, in contrast to other chromosome arms, the gene-rich portion of the 4^th^ chromosome is enriched in HP1a and H3K9me, and here the enrichment within gene bodies depends on Setdb1. The methylation-independent HP1a promoter binding correlates with HP2 and with “open” chromatin structure. We suggest that the methylation-independent and -dependent binding of HP1a are fundamental steps in the transmission, propagation and spreading of this epigenetic mark, hence our observations provide important insights and the basis of a novel model of gene regulation in highly heterochromatic regions.

## Materials and Methods

### Fly stocks and genetic crosses

Flies were cultivated and crossed in vials with potato mash-yeast-agar medium at 25°C. The genotypes used for the experiments were wild type (Oregon R), *Pof^D119^/Pof^D119^* [27], *Su(var)3-9^06^/Su(var)3-9^evo^* [77], *Setdb1^10.1a^/Setdb1^10.1a^* [12] and *G9a^RG5^/G9a^RG5^* [6]. Homozygous mutant third instar larvae were collected from the fly stocks *Df(1)w^67c23^y w; Pof^D119^*, *w; Setdb1^10.1a^*/*CyO GFP* and *w*; *G9a^RG5^*. To generate *trans*-heterozygous *Su(var)3-9^evo^/Su(var)3-9^06^* third instar larvae we crossed *w^m4^; Su(var)3-9^evo^* to *w^m4^; Su(var)3-9^06^/TM6, Tb* and selected the non-*Tb* larvae offspring.

### Chromatin immunoprecipitation

For our ChIP-chip experiments we used salivary glands from third instar larvae. The ChIP experiments were done as previously described [27], [32] using 3 µl of anti-HP1a (PRB291C, Covance), 3 µl of anti-H3K9me2 (ab1220, abcam) and 6 µl of anti-H3K9me3 (ab8898, abcam) for precipitations. We generated two biological replicates with each of three antibodies (anti-HP1a, -H3K9me2 and -H3K9me3) for each of four genotypes: wild type, *Su(var)3-9^evo/06^*, *Setdb1^10.1a^* and *G9a^RG5^*. For *Pof^D119^* we generated two biological replicates with anti-HP1a antibodies. The purified ChIP and input DNA samples were amplified using a WGA2 GenomePlex Complete Whole Genome Amplification kit (Sigma) according to the supplier's recommendations. The amplified DNA was purified with a QIAquick PCR purification kit (Qiagen). To verify that no amplification bias affected the enrichment profiles, we analyzed the ChIP DNA/input DNA ratios before and after the amplifications, using real time PCR as previously described [32].

### Microarray analysis

For tiling array analysis, the amplified ChIP DNA samples were fragmented, labeled and hybridized to an Affymetrix *Drosophila* Genome 2.0 array. In total, 44 DNA samples were hybridized (including ChIP and input samples). The signal intensity data generated were analyzed with Affymetrix Tiling Analysis Software (v. 1.1.02), using 200 bp bandwidth as a parameter only for smoothing and perfect match. The enrichment profiles were produced as ChIP DNA/input DNA ratios expressed in log_2_ scale. The complete data set is available at http://www.ncbi.nlm.nih.gov/geo/ (accession: GSE38366). The occupancy profiles obtained were visualized and analyzed using Integrated Genome Browser (6.2.2).

### Average gene profiles

Average gene profiles were calculated using a custom perl script and *D. melanogaster* genome sequence annotation 5.43. Profiles of HP1a binding and the H3K9me2 and H3K9me3 occupancy of all active genes were calculated for four defined regions: the 4^th^ chromosome, region 2L:31 (previously defined in [26]), the pericentromeric regions of 2L, 2R and 3L (previously defined in [35]) and euchromatic regions, i.e. chromosome arms 2L, 2R, 3L and 3R excluding 2L:31 and pericentromeric regions. The genes within each region were rescaled to the same relative length and the transcripts were divided into five bins. For each gene only the most strongly expressed transcript (deduced from results of RNA-seq analysis of 3^rd^ instar larvae salivary gland tissue [78]) and the corresponding transcription start site (TSS) and exons were used. The promoter was defined as the region extending 500 bp upstream of each TSS or to the next TSS or transcription stop site (if closer). The intergenic region for each gene was defined as the region upstream of the defined promoter to the next TSS or transcription stop site and for each gene average binding and occupancy values for introns were calculated. The average profiles were plotted as average values for the defined intergenic regions followed by the promoter, the five transcript bins and finally the introns.

### Immunostaining of polytene chromosomes

Polytene chromosomes from the salivary glands of wild type, *Su(var)3-9^06^/Su(var)3-9^evo^*, *Setdb1^10.1a^* and *G9a^RG5^* 3^rd^ instar larvae were prepared and stained as previously described [79]. For this, we used anti-HP1a (PRB291C, 1∶400 dilution, Covance) primary antibodies, goat anti-rabbit antibodies conjugated with AlexaFluor555 (1∶300, Molecular Probes) as secondary antibodies and counterstained the squashes with DAPI (1 µg/ml). Images of the squashes were acquired using a Zeiss Axiophot microscope equipped with a KAPPA DX20C CCD camera, then assembled and merged electronically using Adobe Photoshop. For quantitative comparisons of stains, preparations and staining were run in parallel. Nuclei with clear cytology were chosen on the basis of DAPI staining and photographed. At least 20 nuclei for each genotype were used in these comparisons, and at least four slides of each genotype were analyzed.

### Western blotting

For Western blot analysis we dissected salivary glands from third instar larvae in 1× PBS. The salivary glands were homogenized in 2× sample buffer (126 mM Tris-HCl [pH 6.8], 20% glycerol, 4% SDS, 0.005% bromophenol blue, 2.5% β-mercaptoethanol) and heated at 100°C for 10 minutes. The protein samples (from 15 pairs of salivary glands per lane) were separated by SDS-PAGE and transferred to Amersham Hybond-ECL nitrocellulose membrane (GE Healthcare). The membrane was blocked in 5% dry milk in 1× PBS +0.05% Tween-20 overnight at 4°C and incubated with anti-HP1a antibody (PRB291C, Covance) for 2 h at room temperature. HRP-conjugated α-rabbit (Jackson Laboratories) was used as secondary antibody and incubated for 2 h at room temperature. The signal was developed using Super Signal West Dura Extended Duration Substrate (Thermo Scientific) according to the supplier's instructions, and visualized using an LAS 4000 imaging system (Fujifilm). The membrane was reprobed using an anti-α-tubulin antibody (T5168, Sigma) and an HRP-conjugated α-mouse (GE Healthcare) as secondary antibody.

### Sequence motif and overlapping factor searches

To search for enriched motifs in the HP1a promoter peaks of genes active in salivary gland tissue in the 4^th^ chromosome, region 2L:31 and p2L we used the CompleteMOTIFS platform described in [80]. The regions for analysis were defined as 50 bp +/− the HP1a peak center in the promoters. Motif scores were calculated by scanning the identified motif in promoter regions (500 bp upstream of TSS) for the 4^th^ chromosome, region 2L:31, p2L, and the remaining part of chromosome 2L as a control. To look for factors overlapping the HP1a promoter peaks we used the binding data for chromatin factors in S2 cell lines generated by the modENCODE project [14], [81]. In total, 51 unique chromatin factors were included in the analysis, i.e., all chromatin-associated factors mapped in S2 cells by modENCODE (modMine release v20). Fourteen chromatin factors were identified as bound to >50% of chromosome 4 promoters. A binary heat-map was generated by classing all promoters on chromosome 2L and chromosome 4 as either bound or unbound by these factors. To generate average profiles of DNase sensitivity we used the DNase hypersensitivity (DHS) data obtained for embryos, developmental stage 14, from [82]. For each gene only the most strongly expressed transcript (deduced from embryo 22–24 h RNA-seq results [78]) and the TSS and exons were used. To generate average HP2 profiles we used the data from S2 cells provided by modENCODE and the corresponding RNA-seq results [14], [78].

## Supporting Information

Figure S1 HP1a (blue), H3K9me3 (gray) and H3K9me2 (green) profiles for all chromosome arms in salivary gland tissue from wild type.
(PDF)

Figure S2 HP1a and H3K9me profiles in three illustrative regions of 2L:31 and pericentromeric regions, respectively, in wild type and *Setdb1* mutant backgrounds. Numbers along the x-axis denote chromosomal positions along the chromosomes in Mb. The y-axis shows the ChIP enrichment in log_2_ ratios. Genes expressed from left to right and vice versa are shown above and below the horizontal lines, respectively. The HP1a methylation-independent promoter peaks are indicated by yellow boxes. Note that H3K9me in promoters is dependent on Setdb1 also in pericentromeric regions.
(PDF)

Figure S3 Binding profiles of 14 chromatin-associated factors classified as bound to >50% of chromosome 4 promoters. A representative 200 kb region from the 4^th^ chromosome is shown. The enrichment profile of HP1a in *Setdb1* mutants is shown as a reference. Note that this profile is from salivary gland tissue (this study) while the remaining profiles are from S2 cells (modENCODE). The yellow boxes indicate H3K9me-independent promoter peaks (in salivary glands) and the gray box the two genes, *CG1909* and *onecut*, that are unexpressed but bind HP1a independently of POF and Setdb1.
(PDF)

Table S1 Manually annotated promoter peaks on the 4^th^ chromosome enriched in HP1a independently of H3K9me.
(PDF)
