# Supplementary figures and images for: HP1a Recruitment to Promoters Is Independent of H3K9 Methylation in Drosophila melanogaster
Source: PLoS Genet. 2012 Nov 15;8(11):e1003061. doi: 10.1371/journal.pgen.1003061 (PMC3499360; doi:10.1371/journal.pgen.1003061)

Figure S1

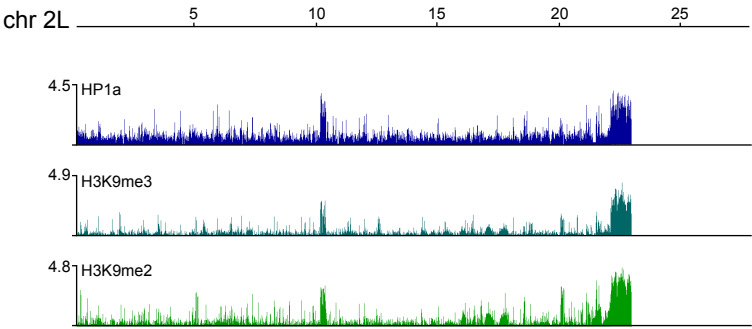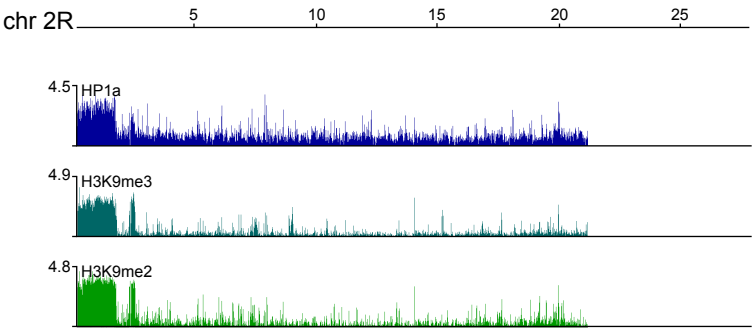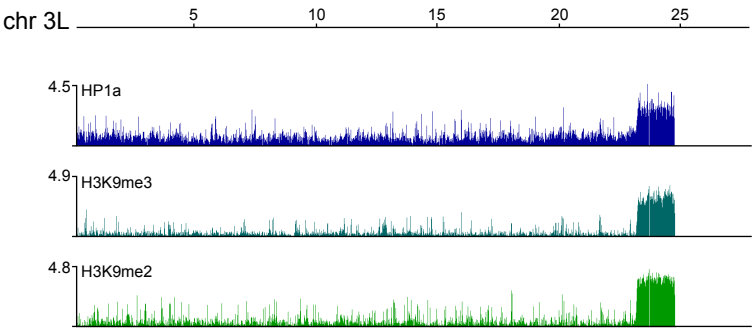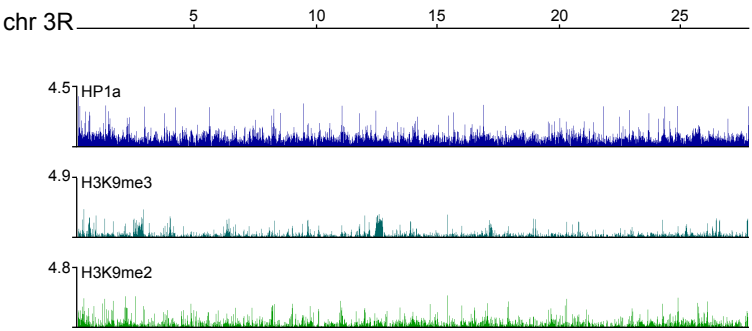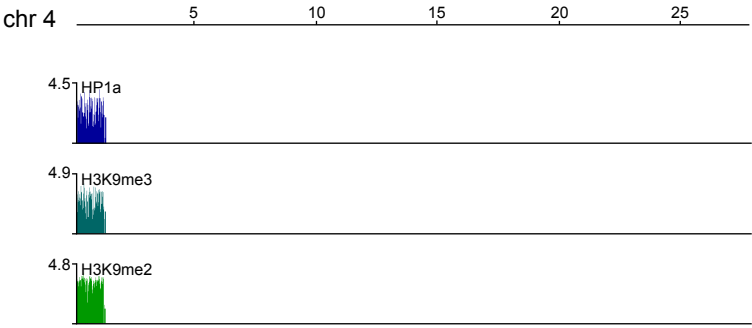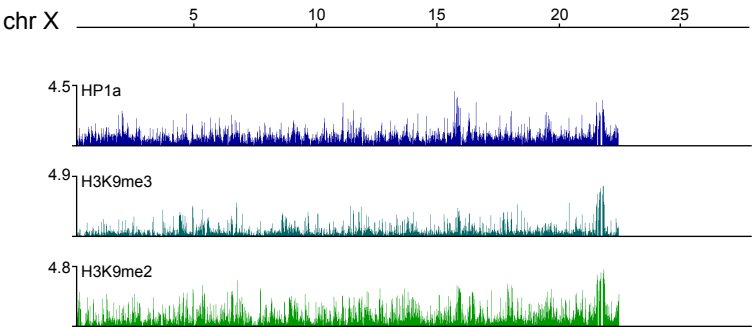

Supplement: Figure S1 — HP1a (blue), H3K9me3 (gray) and H3K9me2 (green) profiles for all chromosome arms in salivary gland tissue from wild type. (PDF) [file pgen.1003061.s001.pdf]

**A**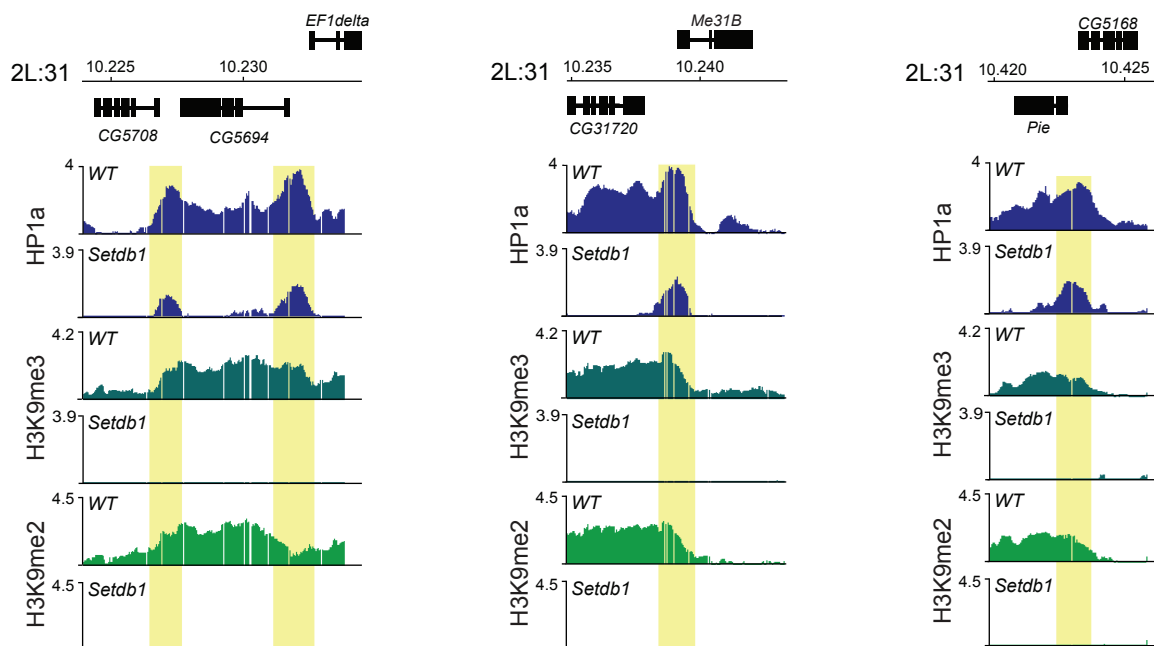**B**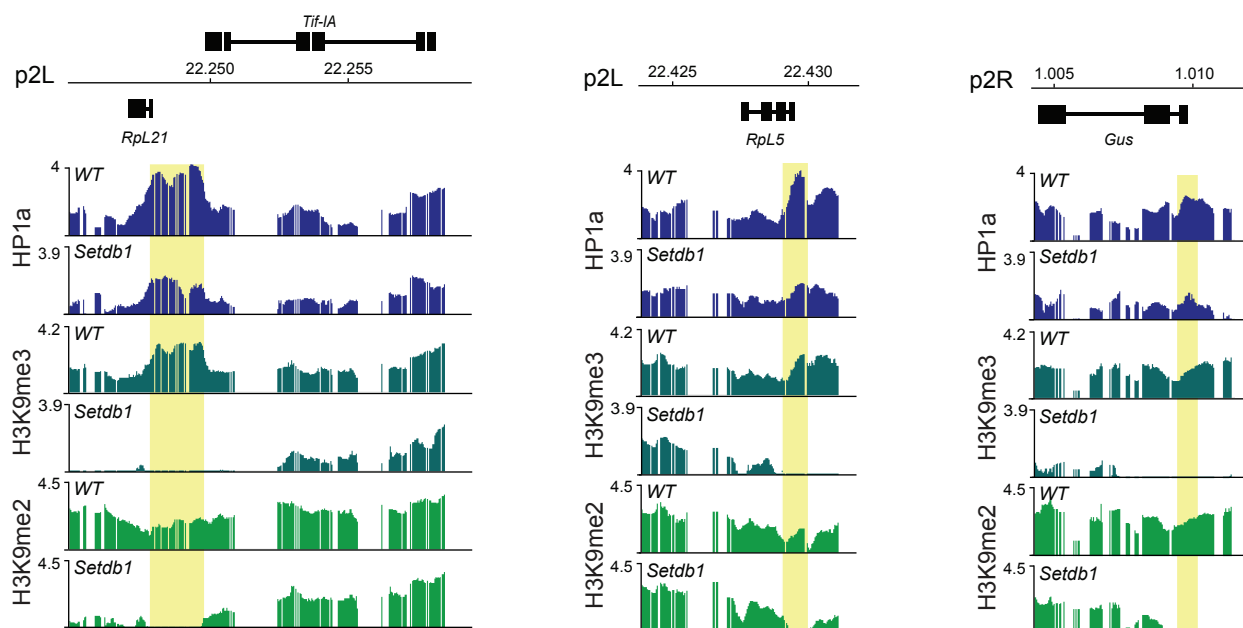

Supplement: Figure S2 — HP1a and H3K9me profiles in three illustrative regions of 2L:31 and pericentromeric regions, respectively, in wild type and Setdb1 mutant backgrounds. Numbers along the x-axis denote chromosomal positions along the chromosomes in Mb. The y-axis shows the ChIP enrichment in log2 ratios. Genes expressed from left to right and vice versa are shown above and below the horizontal lines, respectively. The HP1a methylation-independent promoter peaks are indicated by yellow boxes. Note that H3K9me in promoters is dependent on Setdb1 also in pericentromeric regions. (PDF) [file pgen.1003061.s002.pdf]

Figure S3

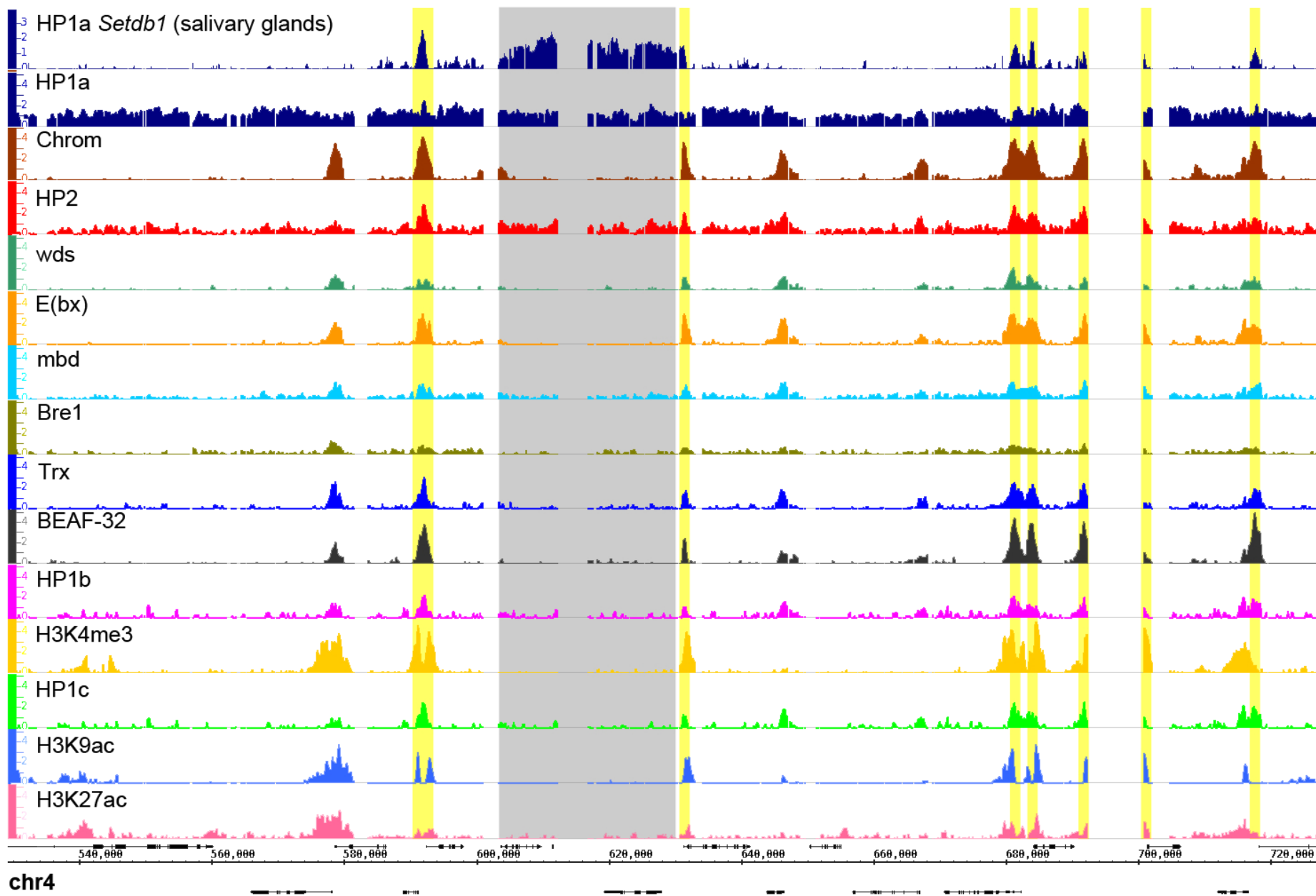

Supplement: Figure S3 — Binding profiles of 14 chromatin-associated factors classified as bound to >50% of chromosome 4 promoters. A representative 200 kb region from the 4th chromosome is shown. The enrichment profile of HP1a in Setdb1 mutants is shown as a reference. Note that this profile is from salivary gland tissue (this study) while the remaining profiles are from S2 cells (modENCODE). The yellow boxes indicate H3K9me-independent promoter peaks (in salivary glands) and the gray box the two genes, CG1909 and onecut, that are unexpressed but bind HP1a independently of POF and Setdb1. (PDF) [file pgen.1003061.s003.pdf]
