# Supplementary material for: HP1a Recruitment to Promoters Is Independent of H3K9 Methylation in Drosophila melanogaster
Source: PLoS Genet. 2012 Nov 15;8(11):e1003061. doi: 10.1371/journal.pgen.1003061 (PMC3499360; doi:10.1371/journal.pgen.1003061)
Supplement: Table S1 — Manually annotated promoter peaks on the 4th chromosome enriched in HP1a independently of H3K9me. (PDF) [file pgen.1003061.s004.pdf]

Table S1

Peak position	Gene	Transcription start site	Strand (+/-)	Peak in salivary glands (1 or 0)	Peak in S2 (1 or 0)	Comments
64 557	plexB	64403	(-)	1	1	
88117	Rps3A	87863	(-)	1	1	or pan in (+)
88707	Rps3A	87863	(-)	1	1	or pan in (+)
150801	Ank	150338	(-)	1	1	
152644	CG32000	152849	(+)	1	1	
200206	CG2219	199980	(-)	1	1	
214715	CG2316	214448	(-)	1	1	several TSS
215746	CG2316	214448	(-)	1	1	several TSS
226787	CG31998	226552	(-)	1	1	
230737	Crk	230920	(+)	1	1	
334709	CG2177	334534	(-)	1	1	
335382	CG32850	335571	(+)	1	1	
380321	Hcf	380602	(+)	1	1	or CG2165 in (-)
465360	CaMKI	466145	(+)	1	1	or lgs in (-)
591887	CG1970	591420	(-)	1	1	or Ephrin in (+)
630954	Eph	631311	(+)	1	1	
6811442	gw	681222	(-)	1	1	several TSS
683925	Slip1	684213	(+)	1	1	
700919	CG11360	701334	(+)	1	1	
717585	myoglianin	716675	(-)	1	1	
808795	MED26	808262	(-)	1	1	
888161	CG11148	887652	(-)	1	1	
951741	eIF-4G	951347	(-)	1	1	
979544	CG32016	978958	(-)	1	1	
980721	CG32016	978958	(-)	1	1	or CG11093 in (+)
1047914	plexA	1047654	(-)	1	1	
1048653	plexA	1047654	(-)	1	1	
1081724	Zyx102EF	1081479	(-)	1	1	
1144536	Arf102F	1144799	(+)	1	1	or cals in (-)
1202512	Pho	1202271	(-)	1	1	
1206325	CG33521	1206430	(+)	1	1	
1217878	CG17471	1217466	(-)	1	1	
1218715	Mitf	1219483	(+)	1	1	
1234875	Dyrk3	1234433	(-)	1	1	
